# Association between dental consultation and oral health status among male Japanese employees

**DOI:** 10.1002/1348-9585.12104

**Published:** 2019-12-21

**Authors:** Yoshihiro Shimazaki, Toshiya Nonoyama, Yoshikazu Miyano, Yasushi Miyata, Kazuaki Hisada, Tsuneyasu Nagasawa

**Affiliations:** ^1^ Department of Preventive Dentistry and Dental Public Health School of Dentistry Aichi Gakuin University Nagoya Japan; ^2^ Kariya Dental Association Kariya Japan

**Keywords:** dental consultation, oral health examination, oral health status, tooth loss, workplace

## Abstract

**Objective:**

To investigate the association between dental consultation and oral health status among male Japanese employees.

**Methods:**

The participants were 3351 male employees who received a workplace oral health examination conducted at the ages of 35, 40, 45, 50, 55, and 59 years before retirement in conjunction with an annual health checkup. Data on dental expenditures were collected from health insurance claims. The number of dental visits and dental care expenses, alone or in combination, were used as indices of the dental consultation status for the analyses. The effects of dental consultation status on oral health status (number of total teeth, number of decayed teeth, and periodontal status) were analyzed using multivariate multinomial logistic regression analyses adjusted for confounders.

**Results:**

Multivariate analyses revealed that the odds ratio (OR) for 20‐27 teeth (losing 1‐8 teeth) was significantly higher (OR 1.4, 95% confidence interval (CI) 1.1‐1.7) in those who had a high number of dental visits and high dental care expenses than in those who did not have a dental visit. By contrast, the ORs for ≤19 teeth (losing ≥9 teeth), having ≥3 decayed teeth, or having a periodontal pocket ≥6 mm were significantly lower (OR 0.2, 95% CI 0.1‐0.6; OR 0.5, 95% CI 0.3‐0.6; OR 0.7, 95% CI 0.5‐1.0, respectively) in those who had fewer dental visits and lower dental care expenses.

**Conclusions:**

These results imply that the dental consultation status is associated with oral health status among male employees.

## 1 INTRODUCTION

The number of total teeth present in Japanese adults has recently increased.^1^ This is thought to be due to various factors, such as improved oral hygiene habits,^1^ advancements in dental materials and dental treatment technology, and an increase in the number of people who undergo dental examinations.^2^ On the other hand, there are health disparities related to oral health in adults, as some adults still lose many teeth.^1^

It is important to prevent dental diseases, such as dental caries and periodontal disease, to prevent the loss of teeth.^3^, ^4^ The most basic element to prevent dental disease is self‐care oral cleaning. It is thought that oral hygiene has a greater effect if correct instruction is provided by dental health experts. In addition, because it is difficult to self‐assess oral health status correctly, regular checks by dental health experts are necessary to maintain oral health. In fact, it has been shown that dental visits to prevent and treat dental diseases suppress tooth loss.^5^

Non‐smokers and people with high health awareness tend to visit the dentist.^6^, ^7^ In addition, an oral health examination plays an important role as an opportunity to visit the dentist. Those who regularly receive voluntary dental health checkups at the workplace have better periodontal status and fewer lost teeth.^8^ Regular dental visits prevent tooth loss.^9^, ^10^ However, the public oral health checkup system for adults in Japan is insufficient.^11^ In addition, it seems that those who need dental treatment as a result of an oral health checkup do not necessarily attend a subsequent dental visit. In Japan, medical insurance covers preventive treatments, such as periodic calculus removal and periodontal maintenance treatment. Under such a medical insurance system, it is meaningful to clarify how dental consultation after dental checkups of workers in the workplace is related to their oral health.

Many studies have investigated dental consultation visits using questionnaires.^12^, ^13^ If it is possible to grasp dental consultation status, including the number of dental visits and the required dental care costs based on medical insurance data, it may be possible to clarify the association between the treatment cost burden due to dental consultation and the benefits obtained. In particular, it would be desirable to demonstrate the effectiveness of undergoing dental treatments on the prevention and management of tooth decay and periodontal disease, which are relatively inexpensive. In this study, we studied dental consultation status from dental insurance claims data for those who received oral health examinations in the workplace, and analyzed the association between this status and oral health conditions to examine the effects of dental consultation on oral health maintenance.

## 2 SUBJECTS AND METHODS

### 2.1 Study design and participants

This cross‐sectional study was conducted on male employees working for a company in the Toyota Group in Kariya, Aichi Prefecture. The company conducts an annual health checkup for all employees based on the Industrial Safety and Health Act. In addition, oral health examinations are conducted at the ages of 35, 40, 45, 50, 55, and 59 years in conjunction with the annual health checkup. The Kariya Dental Association and another private health‐care provider were responsible for half of the oral health examinations. Overall, 7467 employees underwent an oral health examination between April 2008 and March 2013. This study targeted 3549 employees who underwent oral health examinations by the Kariya Dental Association, of whom 7 who were edentulous and 191 who lacked data for the analysis were excluded; 3351 were finally included in the study. The characteristics of the participants are shown in Table 1.

**Table 1:** Characteristics of the study participants

Variable	N (%)
Age (years)	
35	844 (25.2)
40	882 (26.3)
45	715 (21.3)
50	471 (14.1)
55	217 (6.5)
59	222 (6.6)
Smoking status	
Non‐smoker	1625 (48.5)
Smoker	1726 (51.5)
Number of teeth present	
≥28	2395 (71.5)
20‐27	889 (26.5)
≤19	67 (2.0)
Number of decayed teeth	
0	2048 (61.1)
1‐2	802 (23.9)
≥3	501 (15.5)
CPI code	
0‐2	2934 (87.6)
3	183 (5.5)
4	234 (7.0)
Dental consultation status	
Number of visits (days)	
None (0)	1702 (50.8)
Low (1‐4)	835 (24.9)
High (≥5)	814 (24.3)
Care expenses (yen)	
None (0)	1702 (50.8)
Low (<30 000)	881 (26.3)
High (≥30 000)	768 (22.9)
Number of visit × Care expenses	
None × None	1702 (50.8)
Low × Low	773 (23.1)
Low × High	62 (1.9)
High × Low	108 (3.2)
High × High	706 (21.1)

Variable	Mean (SD)
Body mass index (kg/m^2^)	23.9 (3.3)
Number of teeth present	27.3 (3.2)
Number of sound teeth	14.0 (5.9)
Number of decayed teeth	1.1 (2.2)
Number of filled teeth	12.1 (5.2)
Number of missing teeth	0.9 (1.9)

Abbreviations: CPI, community periodontal index; N, number of subjects; SD, standard deviation.

Health examinations were not conducted for the survey, but rather for periodic health checkups for the employees, so we did not obtain consent from the participants. We obtained permission from the insurer to use the de‐identified data for this study, and this study was approved by the Institutional Review Board of Aichi Gakuin University, School of Dentistry, and was conducted in full accordance with the World Medical Association Declaration of Helsinki.

### 2.2 Measures

The oral health examination was conducted throughout the year by trained dentists who were members of the Kariya Dental Association using a manual based on the standardized criteria for an oral examination.^14^ The checkup was conducted as part of a general health checkup. Tooth status was recorded as sound, decayed, filled, or missing, and the number of total teeth was the total number of sound, decayed, and filled teeth including third molars. The community periodontal index (CPI) was used to assess periodontal status^14^ because this method is commonly used in public oral health examinations in Japan. The oral cavity of each participant was divided into six sextants. The index tooth numbers were 11, 16, 17, 26, 27, 31, 36, 37, 46, and 47. Measurements were made at six sites (mesiobuccal, mid‐buccal, distobuccal, distolingual, mid‐lingual, and mesiolingual) on each index tooth. The CPI scores were coded as follows: healthy (code 0), bleeding after probing (code 1), dental calculus detected by probing (code 2), 4‐5 mm shallow pocket (code 3), ≥6 mm deep pocket (code 4), and relevant tooth missing (code X).

Height and body weight were measured to calculate body mass index (kg/m^2^). Smoking habit (non‐smoker/smoker) was queried using a self‐reported questionnaire.

### 2.3 Classification of the dental consultation

**Figure 1:**
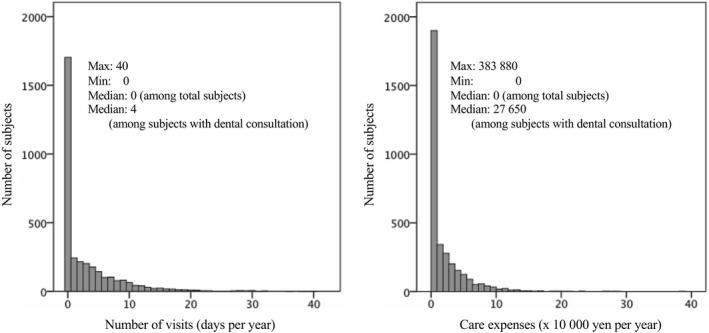
Distribution of dental consultation status (number of visits, care expenses)

Data on dental expenditures were collected for each individual from health insurance claims submitted between April 2008 and March 2013. To assess dental consultation status, we examined the number of visits and care expenses for the year in which the subjects received an oral health examination based on the social insurance expenditure data. Figure 1 shows the distribution of dental consultations (assessed by the number of visits and the care expenses). Approximately half of the subjects did not visit a dental clinic for 1 year. In this study, we used three dental consultation parameters to examine the association between dental consultation status and oral health status in more detail: the number of dental visits, dental care expenses, and their combination. Based on the median number of dental visits, the subjects were further classified as having a low (1‐4 days) or high (≥5 days) number of dental visits. In addition, annual dental care expenses were similarly classified into low (<30 000 yen) and high (≥30 000 yen) costs.

### 2.4 Statistical analyses

Continuous variables are presented as mean with standard deviation, and were assessed using analysis of variance followed by post hoc Bonferroni correction. Categorical data are expressed as numbers and percentages (%), and were compared using the chi‐square test.

The number of total teeth, number of decayed teeth, and CPI code were each divided into three groups for the analysis: ≥28, 20‐27, and ≤19 teeth; 0, 1‐2, and ≥3 teeth; and 0‐2, 3, and 4, respectively. Multinomial logistic regression analyses were performed to calculate odds ratios (ORs) and 95% confidence intervals (CIs) for the effects of dental consultation status and other variables on the number of total teeth, number of decayed teeth, and CPI code. Age, smoking status, body mass index, and dental consultation status data that were marginally significant in bivariate analyses (*P* < .1) were included as independent variables in multivariate multinomial logistic regression analyses. Statistical analyses were carried out using SPSS 24.0 software (IBM). A *P* < .05 was considered significant.

## 3 RESULTS

The comparison of oral health status according to dental consultation status is shown in Table 2. The number of total teeth was the highest, and the numbers of decayed teeth and missing teeth were the lowest, in participants who visited a dental clinic 1‐4 times/year, and their annual dental care expenses were <30 000 yen. Those who did not receive dental treatment for 1 year had a high number of decayed and missing teeth.

**Table 2:** Comparison of tooth status according to dental consultation status

	Dental consultation status, mean (SD)					*P*‐value^a^
	Number of visits (days) Care expenses (yen)					
	None (0) None (0) N = 1702	Low (1‐4) Low (<30 000) N = 773	Low (1‐4) High (≥30 000) N = 62	High (≥5) Low (<30 000) N = 108	High (≥5) High (≥30 000) N = 706	
Number of teeth present	27.9 (3.1)^b^	28.1 (2.2)^c^	27.6 (3.2)	27.7 (4.2)	27.3 (3.2)^b,c^	<.001
Number of sound teeth	16.8 (6.4)^b,d^	16.3 (5.7)^c^	14.1 (5.8)^d^	15.8 (6.1)	14.0 (5.9)^b,c^	<.001
Number of decayed teeth	1.6 (3.2)^e,f^	0.7 (1.5)^e,g^	0.8 (1.9)	1.0 (2.3)	1.1 (2.2)^f,g^	<.001
Number of filled teeth	9.6 (5.4)^b,e,h^	11.1 (5.1)^e,i^	12.7 (5.4)^h^	11.0 (5.6)	12.1 (5.2)^b,i^	<.001
Number of missing teeth	0.9 (2.2)^j^	0.6 (1.2)^i,j^	0.9 (2.5)	0.5 (2.1)	0.9 (1.9)^i^	<.001

Abbreviations: N, number of subjects; SD, standard deviation.

^b‐j^The same alphabet symbol is attached to the group where the significant difference was recognized in the post hoc comparisons (Bonferroni correction).

^g^ *P* < .05.

^d,f,i,j^ *P* < .01.

^b,c,e,h^ *P* < .001.

a One‐way analysis of variance.

Univariate and multivariate multinomial logistic regression analyses were performed to investigate the magnitude of risk and the effect of the dental consultation status independently of other factors on the number of total teeth (Table 3). The ORs for loss of 1‐8 teeth (20‐27 teeth) were significantly higher in those who had ≥5 days of dental visits and ≥30 000 yen of dental care expenses (OR 1.4, 95% CI 1.1‐1.7) than in those who did not have a dental visit. On the other hand, the ORs for loss of ≥9 teeth (≤19 teeth) were significantly lower in those who had 1‐4 dental consultation days and <30 000 yen of dental care expenses (OR 0.2, 95% CI 0.1‐0.6).

**Table 3:** Association of dental consultation status with the number of total teeth determined by univariate and multivariate multinomial logistic regression analyses

	Number of teeth present			Dependent variable: number of teeth present		Dependent variable: number of teeth present	
	≥28	20‐27	≤19	20‐27 vs ≥28	≤19 vs ≥28	20‐27 vs ≥28	≤19 vs ≥28
	N = 2395	N = 889	N = 67	Crude OR (95% CI)	Crude OR (95% CI)	Adjusted OR^a^ (95% CI)	Adjusted OR^a^ (95% CI)
Dental consultation status (number of visits, day)							
None (0)	1248 (52.1)	418 (47.0)	36 (53.7)	1	1	1	1
Low (1‐4)	618 (25.8)	211 (23.7)	6 (9.0)	1.0 (0.8‐1.2)	0.3 (0.1‐0.8)*	0.9 (0.8‐1.2)	0.3 (0.1‐0.7)**
High (≥5)	529 (22.1)	260 (29.2)	25 (37.3)	1.5 (1.2‐1.8)***	1.6 (1.0‐2.8)	1.3 (1.0‐1.5)*	1.1 (0.6‐1.9)
Dental consultation status (care expenses, yen)							
None (0)	1248 (52.1)	418 (47.0)	36 (53.7)	1	1	1	1
Low (<30 000)	663 (27.7)	210 (23.6)	8 (11.9)	0.9 (0.8‐1.1)	0.4 (0.2‐0.9)*	0.9 (0.7‐1.0)	0.3 (0.2‐0.8)**
High (≥30 000)	484 (20.2)	261 (29.4)	23 (34.3)	1.6 (1.3‐1.9)***	1.6 (1.0‐2.8)	1.4 (1.1‐1.7)***	1.1 (0.6‐2.0)
Dental consultation status (number of visits × care expenses)							
None × None	1248 (52.1)	418 (47.0)	36 (53.7)	1	1	1	1
Low × Low	581 (24.3)	188 (21.1)	4 (6.0)	1.0 (0.8‐1.2)	0.2 (0.1‐0.7)**	0.9 (0.7‐1.1)	0.2 (0.1‐0.6)**
Low × High	37 (1.5)	23 (2.6)	2 (3.0)	1.9 (1.1‐3.2)*	1.9 (0.4‐8.1)	1.6 (0.9‐2.8)	1.6 (0.3‐7.8)
High × Low	82 (3.4)	22 (2.5)	4 (6.0)	0.8 (0.5‐1.3)	1.7 (0.6‐4.9)	0.7 (0.4‐1.1)	1.1 (0.4‐3.5)
High × High	447 (18.7)	238 (26.8)	21 (31.3)	1.6 (1.3‐1.9)***	1.6 (0.9‐2.8)	1.4 (1.1‐1.7)**	1.1 (0.6‐1.9)

Abbreviations: CI, confidence interval; OR, odds ratio.

a Adjusted for age, smoking status and body mass index.

* *P* < .05.

** *P* < .01.

*** *P* < .001.

Table 4 shows the association between dental consultation status and the number of decayed teeth according to univariate and multivariate multinomial logistic regression analyses. Employees with 1‐4 dental consultation days had a significantly lower OR for having 1 or 2 decayed teeth (OR 0.8, 95% CI 0.7‐1.0), and those with any dental consultations (≥1 dental consultation days and >0 yen of dental care expenses) had a significantly lower OR for having ≥3 decayed teeth. For the dental consultation status, the OR for having ≥3 decayed teeth was significantly lower in those who had few 1‐4 dental consultation days and <30 000 yen of dental care expenses (OR 0.5, 95% CI 0.3‐0.6).

**Table 4:** Association of dental consultation status with the number of decayed teeth determined by univariate and multivariate multinomial logistic regression analyses

	Number of decayed teeth			Dependent variable: number of decayed teeth		Dependent variable: number of decayed teeth	
	0	1‐2	≥3	1‐2 vs 0	≥3 vs 0	1‐2 vs 0	≥3 vs 0
	N = 2048	N = 802	N = 501	Crude OR (95% CI)	Crude OR (95% CI)	Adjusted OR^a^ (95% CI)	Adjusted OR^a^ (95% CI)
Dental consultation status (number of visits, day)							
None (0)	990 (48.3)	401 (50.0)	311 (62.1)	1	1	1	1
Low (1‐4)	576 (28.1)	182 (22.7)	77 (15.4)	0.8 (0.6‐1.0)*	0.4 (0.3‐0.6)***	0.8 (0.7‐1.0)*	0.5 (0.3‐0.6)***
High (≥5)	482 (23.5)	219 (27.3)	113 (22.6)	1.1 (0.9‐1.4)	0.7 (0.6‐1.0)*	1.1 (0.9‐1.4)	0.8 (0.6‐1.0)*
Dental consultation status (care expenses, yen)							
None (0)	990 (48.3)	401 (50.0)	311 (62.1)	1	1	1	1
Low (<30 000)	604 (29.5)	194 (24.2)	83 (16.6)	0.8 (0.7‐1.0)*	0.4 (0.3‐0.6)***	0.8 (0.7‐1.0)	0.5 (0.4‐0.6)***
High (≥30 000)	454 (22.2)	207 (25.8)	107 (21.4)	1.1 (0.9‐1.4)	0.8 (0.6‐1.0)*	1.1 (0.9‐1.4)	0.8 (0.6‐1.0)*
Dental consultation status (number of visits × care expenses)							
None × None	990 (48.3)	401 (50.0)	311 (62.1)	1	1	1	1
Low × Low	534 (26.1)	169 (21.1)	70 (14.0)	0.8 (0.6‐1.0)*	0.4 (0.3‐0.6)***	0.8 (0.7‐1.0)	0.5 (0.3‐0.6)***
Low × High	42 (2.1)	13 (1.6)	7 (1.4)	0.8 (0.4‐1.4)	0.5 (0.2‐1.2)	0.8 (0.4‐1.4)	0.5 (0.2‐1.2)
High × Low	70 (3.4)	25 (3.1)	13 (2.6)	0.9 (0.6‐1.4)	0.6 (0.3‐1.1)	0.9 (0.6‐1.5)	0.6 (0.3‐1.2)
High × High	412 (20.1)	194 (24.2)	100 (20.0)	1.2 (0.9‐1.4)	0.8 (0.6‐1.0)*	1.2 (0.9‐1.4)	0.8 (0.6‐1.0)

Abbreviations: CI, confidence interval; OR, odds ratio.

a Adjusted for age, smoking status, and body mass index.

* *P* < .05.

*** *P* < .001.

Table 5 shows the association between the dental consultation status and the CPI code. The OR for a CPI code of 3 (4‐5 mm periodontal pocket) was significantly higher in those with ≥5 dental consultation days (OR 1.5, 95% CI 1.0‐2.1), whereas the OR for a CPI code of 4 (≥6 mm periodontal pocket) was significantly lower in those who had 1‐4 dental consultation days (OR 0.6, 95% CI 0.4‐0.9) and <30 000 yen of dental care expenses (OR 0.6, 95% CI 0.5‐0.9), and the combination of these (OR 0.7, 95% CI 0.5‐1.0) compared with those with no dental visits.

**Table 5:** Association of dental consultation status with the CPI code determined by univariate and multivariate multinomial logistic regression analyses

	CPI code			Dependent variable: CPI code		Dependent variable: CPI code	
	0‐2	3	4	3 vs 0‐2	4 vs 0‐2	3 vs 0‐2	4 vs 0‐2
	N = 2934	N = 183	N = 234	Crude OR (95% CI)	Crude OR (95% CI)	Adjusted OR^a^ (95% CI)	Adjusted OR^a^ (95% CI)
Dental consultation status (number of visits, day)							
None (0)	1491 (50.8)	83 (45.4)	128 (54.7)	1	1	1	1
Low (1‐4)	755 (25.7)	39 (21.3)	41 (17.5)	0.9 (0.6‐1.4)	0.6 (0.4‐0.9)*	0.9 (0.6‐1.4)	0.6 (0.4‐0.9)*
High (≥5)	688 (23.4)	61 (33.3)	65 (27.8)	1.6 (1.0‐2.2)**	1.1 (0.8‐1.5)	1.5 (1.0‐2.1)*	1.0 (0.7‐1.3)
Dental consultation status (care expenses, yen)							
None (0)	1491 (50.8)	83 (45.4)	128 (54.7)	1	1	1	1
Low (<30 000)	792 (27.0)	45 (24.6)	44 (18.8)	1.0 (0.7‐1.5)	0.6 (0.5‐0.9)*	1.0 (0.7‐1.5)	0.7 (0.5‐0.9)*
High (≥30 000)	651 (22.2)	55 (30.1)	62 (26.5)	1.5 (1.1‐2.2)*	1.1 (0.8‐1.5)	1.4 (1.0‐2.0)	1.0 (0.7‐1.3)
Dental consultation status (number of visits × care expenses)							
None × None	1491 (50.8)	83 (45.4)	128 (54.7)	1	1	1	1
Low × Low	698 (23.8)	36 (19.7)	39 (16.7)	0.9 (0.6‐1.4)	0.7 (0.5‐0.9)*	0.9 (0.6‐1.4)	0.7 (0.5‐1.0)*
Low × High	57 (1.9)	3 (1.6)	2 (0.9)	0.9 (0.3‐3.1)	0.4 (0.1‐1.7)	0.8 (0.2‐2.6)	0.3 (0.1‐1.5)
High × Low	94 (3.2)	9 (4.9)	5 (2.1)	1.7 (0.8‐3.5)	0.6 (0.2‐1.6)	1.6 (0.8‐3.4)	0.6 (0.2‐1.4)
High × High	594 (20.2)	52 (28.4)	60 (25.6)	1.6 (1.1‐2.3)*	1.2 (0.9‐1.6)	1.4 (1.0‐2.1)	1.0 (0.7‐1.4)

Abbreviations: CI, confidence interval; CPI, community periodontal index; OR, odds ratio.

a Adjusted for age, smoking status, and body mass index.

* *P* < .05.

** *P* < .01.

## 4 DISCUSSION

We analyzed the association between dental consultation status and oral health status in men who received an oral health checkup at their workplace. Participants who had few dental visits and low annual dental expenses for 1 year had fewer decayed and missing teeth than those who did not have a dental consultation. As we examined the dental consultation status for the year in which subjects received an oral health checkup, some participants had dental visits because they required dental treatment as a result of their oral health checkups. Although it was unclear whether the time that they received dental treatment was before or after the oral health checkup, participants who had dental treatment had fewer untreated caries. On the other hand, the fact that the number of missing teeth in participants who had received dental treatment was small, they would not be affected by the results of an oral health checkup or the timing of the dental consultation.

Among the participants who visited a dental clinic, those with a high number of consultation days and treatment cost had few teeth or many missing teeth. The number of treatment days and dental care expenses were high as a result of those who had many lost teeth and received prosthetic treatment. Multivariate analysis revealed that participants who had a large number of consultation days and high treatment costs had a higher risk of losing some teeth than those who did not visit a dental clinic. On the other hand, in multivariate analysis, the risk of losing many teeth was significantly lower for those with fewer dental visits and lower treatment costs compared to those who did not visit the dentist. This group included many people who had a dental consultation for the purpose of initial treatment of dental caries and periodic management of periodontal disease. Indeed, those who had <30 000 yen of dental care expenses and 1‐4 days of dental visits were at low risk of having ≥3 untreated teeth and ≥6‐mm periodontal pockets. This implies that the number of visits to the dentist influences oral health status.

We analyzed the number of dental consultation days and dental care expenses from medical insurance claims, so it was possible to accurately grasp dental consultation status. On the other hand, it was unclear what kind of dental treatment the subjects specifically received, as we could not obtain information on disease names or treatment details at the time of the dental consultation. The average number of consultation days for subjects with 1‐4 days of dental consultation and annual treatment expenses <30 000 yen was 2.2 days, and the average treatment expense was about 6800 yen. In a study that compared the average costs of supportive periodical care among several countries, the Japanese data indicated four annual consultation days, and the treatment cost per day was about 3000 yen.^15^ In our study, those who received periodic management of periodontal disease and those who received initial caries treatment were included in the categories of low dental consultation days and treatment costs.

In the company used for this research, an oral health examination was adopted as one of the medical examination items for people every 5 years. Therefore, an oral health checkup at the workplace is considered a trigger for dental consultation by identifying a dental disease without subjective symptoms. Many studies have shown that regular dental visits reduce the risk of tooth loss.^9^, ^10^, ^16^, ^17^ In addition, subjects who frequently receive a dental checkup in the workplace have good periodontal health.^8^ Oral health checkups in the workplace can lead to early treatment of dental caries and management of periodontal disease and thus suppresses tooth loss in working adults.

As the participants in this study were <60 years old, the percentage of those with <20 teeth was low. However, early loss of many teeth is a serious risk factor affecting eating habits^18^ and general health, including medical care costs,^19^, ^20^ and it is feared that this leads to health disparities toward old age. In Health Japan 21 (the second term) advocated by the Ministry of Health, Labor, and Welfare in Japan, extending a healthy life expectancy and reducing health disparities are basic goals of National Health Promotion, and oral health is included as an important lifestyle habit to maintain general health.^21^ It is thought that having dental consultations to prevent loss of teeth will help prolong the healthy life expectancy and reduce health disparities.

Periodontal disease is an important risk factor for tooth loss.^22^, ^23^ Among Japanese adults, the percentage of those who lose teeth due to periodontal disease is high.^4^ In this study, an adequate dental consultation status was associated with a lower risk of advanced periodontitis, which may have resulted in the reduced risk of losing many teeth. Smoking is an important risk factor for periodontal disease,^24^ and many studies showed that smokers lose many teeth.^25^ Indeed, we found that smoking was significantly associated with a risk of having fewer teeth, untreated caries, or deep periodontal pockets (data not shown). As about half of the male employees in this study were smokers, it is important to promote smoking cessation education in the workplace, not only for oral health but also for general health management.

Several limitations to this study should be mentioned. As the oral health checkup in the workplace and the dental consultation year were the same, it remains unclear whether the dental consultation occurred before or after the oral health checkup. Therefore, it is not possible to show the association between an oral health checkup and the dental consultation. However, a certain percentage of people are considered to have a dental visit based on the results of the oral health checkup, and for those who need dental treatment, oral health checkup at the workplace seems to be a trigger for dental consultation. As this was a cross‐sectional study, we could not confirm the presence or absence of tooth loss and the suppressive effects of tooth loss due to dental consultation status. The oral health checkup in this study was not conducted for research, but was conducted for employee health management. As the oral examinations were not calibrated, it was not possible to confirm inter‐examiner reliability, particularly for the periodontal examination. However, errors among examiners were considered not to strengthen the association that we wanted to clarify. Oral health behaviors, such as the frequency of brushing teeth and use of secondary oral hygiene products, are closely related to oral health, but we could not obtain information on oral health behaviors in this study. In addition, this study lacks information on important factors affecting various health conditions, such as socioeconomic status including education level and occupation. Since these factors are thought to be related to dental consultation and oral health statuses, it will be important to consider factors such as oral health behavior and socioeconomic status in future research.

Conducting an oral health checkup in the workplace is a meaningful opportunity to grasp oral health status. However, a checkup is just an evaluation of health status, and a dental consultation is required, if necessary. Regular dental visits are effective for maintaining the health of teeth and periodontal tissues, and to suppress tooth loss. The effects of maintaining oral health through dental visits needs to be further clarified in longitudinal studies.

In conclusion, male adults who had a dental consultation in the workplace had fewer decayed and missing teeth. In particular, those with fewer dental visits and low dental care costs had lower risks of losing many teeth, having untreated dental caries, and having deep periodontal pockets, suggesting that dental consultations are associated with oral health status among male employees.

## DISCLOSURE

*Approval of the research protocol*: This study was approved by the Institutional Review Board of Aichi Gakuin University, School of Dentistry, and was conducted in full accordance with the World Medical Association Declaration of Helsinki. *Informed consent*: Health examinations were not conducted for the survey, but rather for periodic health checkups for the employees, so we did not obtain consent from the participants. We obtained permission from the insurer to use the de‐identified data for this study. *Registry and the registration no. of the study/trial*: N/A. *Animal Studies*: N/A. *Conflict of interest*: None declared.

## AUTHOR CONTRIBUTIONS

YS conceived the ideas; YM, YM, KH, and TN collected the data; YS and TN analyzed the data; and YS led the writing.
